# III-Nitride Magnetron Sputter Epitaxy on Si: Controlling Morphology, Crystal Quality, and Polarity Using Al Seed Layers

**DOI:** 10.1021/acsami.4c03112

**Published:** 2024-06-17

**Authors:** Katrin Pingen, Niklas Wolff, Zahra Mohammadian, Per Sandström, Susanne Beuer, Elizabeth von Hauff, Lorenz Kienle, Lars Hultman, Jens Birch, Ching-Lien Hsiao, Alexander M. Hinz

**Affiliations:** † Fraunhofer Institute for Organic Electronics, Electron Beam and Plasma Technology, Winterbergstrasse 28, D-01277 Dresden, Germany; ‡ Institute of Solid State Electronics, Technische Universität Dresden, Mommsenstrasse 15, D-01069 Dresden, Germany; § Synthesis and Real Structure, Department of Material Science, Kiel University, Kaiserstrasse 2, D-24143 Kiel, Germany; ∥ Kiel Nano, Surface and Interface Science, Kiel University, Christian-Albrechts-Platz 4, D-24118 Kiel, Germany; ⊥ Department of Physics, Chemistry and Biology, Linköpings Universitet, SE-581 83 Linköping, Sweden; # Fraunhofer Institute for Integrated Systems and Device Technology, Schottkystrasse 10, D-91058 Erlangen, Germany

**Keywords:** AlN, GaN, magnetron sputter epitaxy, polarity, Al seed layer

## Abstract

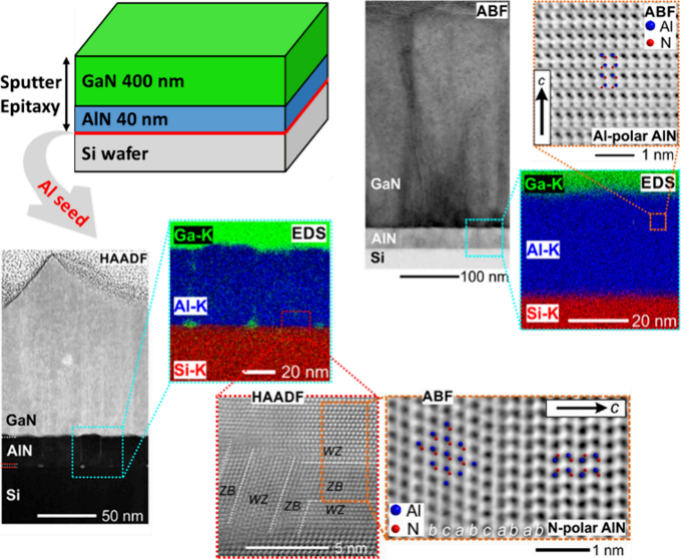

Group III-nitride semiconductors have been subject of intensive research, resulting in the maturing of the material system and adoption of III-nitrides in modern optoelectronics and power electronic devices. Defined film polarity is an important aspect of III-nitride epitaxy as the polarity affects the design of electronic devices. Magnetron sputtering is a novel approach for cost-effective epitaxy of III-nitrides nearing the technological maturity needed for device production; therefore, control of film polarity is an important technological milestone. In this study, we show the impact of Al seeding on the AlN/Si interface and resulting changes in crystal quality, film morphology, and polarity of GaN/AlN stacks grown by magnetron sputter epitaxy. X-ray diffraction measurements demonstrate the improvement of the crystal quality of the AlN and subsequently the GaN film by the Al seeding. Nanoscale structural and chemical investigations using scanning transmission electron microscopy reveal the inversion of the AlN film polarity. It is proposed that N-polar growth induced by Al seeding is related to the formation of a polycrystalline oxygen-rich AlN interlayer partially capped by an atomically thin Si-rich layer at the AlN/Si interface. Complementary aqueous KOH etch studies of GaN/AlN stacks demonstrate that purely metal-polar and N-polar layers can be grown on a macroscopic scale by controlling the amount of Al seeding.

## Introduction

Extensive research efforts dedicated to III-nitride semiconductors have led to the broad integration of GaN and AlN in contemporary optoelectronic and power electronic devices.^1−5^ Established growth methods for III-nitrides, such as metal–organic chemical vapor phase epitaxy (MOVPE), provide high-quality films; however, they rely on high growth temperatures and expensive precursors.^6−11^ In recent years, there has been an increasing focus on exploring alternative growth techniques to enable cost-effective and high-quality manufacturing of electronic devices for widespread applications. Magnetron sputter epitaxy (MSE) is an emerging technology that offers many advantages for device-quality III-nitride growth: cost-effectiveness, high-throughput, and ease of scalability. Sputtering from a solid Ga target enables a stable process and high growth rates for producing high-quality GaN.^12^ Energetic ion bombardment enables lower growth temperatures leading to lower thermal stresses in the films and facilitates direct integration with Si complementary metal-oxide-semiconductor technology. The heteroepitaxial growth on cost-effective Si(111) substrates is the preferred choice for power electronic devices; however, GaN-on-Si epitaxy is characterized by large mismatches in lattice parameter and thermal expansion coefficient. High defect densities and cracking of films exceeding 1 μm in thickness are related issues that still need to be solved in the epitaxy process.^13,14^ A process window for high-quality growth of GaN/AlN by MSE has already been demonstrated.^15−18^ Careful design of buffer layers and strain management is necessary to successfully grow high-quality and crack-free GaN/AlN on Si.^8,14,19^ The integration of an Al seed layer into the process flow has proven beneficial to improve the crystal quality of AlN-on-Si grown by various epitaxy technologies. Enhanced surface diffusion and avoiding the formation of SiN_*x*_ layers promote the adhesion and subsequent growth of AlN layers.^20−29^ However, a thorough investigation of the impact of this seed layer on the film morphology, structural quality, and film polarity of sputtered GaN/AlN/Si(111) stacks has rarely been reported. For wurtzite-type III-nitride-based electronic devices, such as high electron mobility transistors (HEMTs), the polarization direction along the *c*-axis is a key property that must be controlled reliably as it determines the direction of the internal electrical field and thus the device design. While N-polar devices are feasible, metal-polar devices are more common as they are more compatible with standard device fabrication processes and tend to incorporate less oxygen than N-polar films, making it easier to maintain the permitted oxygen impurity levels in lateral HEMT structures.^30−32^ In the future, low impurity levels in combination with high growth rates might make MSE also attractive for vertical power devices based on AlN-rich AlGaN or pure AlN. Apart from defined and homogeneous polarity, a high crystal quality, i.e., low defect densities, and, especially for lateral devices, sharp interfaces are key properties that determine device performance. In this study, polarity control of all-sputtered epitaxial GaN thin films is achieved by seeding Si(111) substrates with Al before growth of an AlN nucleation layer. The impact of the Al seeding on the AlN nucleation and the polarity of the GaN/AlN film stack is studied in detail. The film stacks are characterized by a combination of X-ray diffraction (XRD), etching experiments, and scanning transmission electron microscopy (STEM) paired with chemical analysis via energy-dispersive X-rays spectroscopy (EDS) and electron energy loss spectroscopy (EELS). The growth of the GaN films is realized by MSE from a solid Ga target using an optimized process window described in ref (12).

## Experimental Section

AlN films are grown on approximately 10 mm × 10 mm pieces of Si(111) wafers by reactive direct current magnetron sputtering in an ultrahigh vacuum (UHV) chamber with a base pressure lower than 1 × 10^–6^ Pa. Prior to growth, the n-type Si(111) substrates are chemically etched with aqueous HF to remove the native surface oxide. The substrate holder rotates during deposition to ensure a homogeneous coating of the substrate. The growth temperature is 800 °C, determined by pyrometry calibration, and the total pressure during growth is 0.67 Pa with an N_2_/Ar ratio of 0.3. The sputter chamber is equipped with a confocal Al magnetron in sputter-up configuration that is supplied with 150 W DC power resulting in an AlN growth rate of 200 nm/h. Al seed layer growth times from 0 to 240 s are used to study the influence of different amounts of seeding on the growth of subsequently grown AlN and GaN films. The nominal deposition rate of Al at room temperature amounts to ∼300 nm/h. Since the Al seed layer is not a continuous film, the deposition time instead of a nominal film thickness is used in the description of the experiments. AlN/Si(111) templates with selected Al seed layer growth times are overgrown with GaN without a break in vacuum. GaN sputtering is carried out in a sputter-up configuration using an UHV-magnetron with bespoke cooling designed by PVD Product, Inc. enabling GaN MSE with a solid Ga target.^12^ GaN growth is performed at a total pressure of 0.4 Pa with a partial pressure ratio of N_2_/Ar of 0.3 at a growth temperature of 800 °C on a rotating substrate. Applying a target voltage of 50 W DC power results in a growth rate of 800 nm/h. The crystal structure of the samples is analyzed by high-resolution XRD (Malvern Panalytical Empyrean). As a measure of the crystal quality the full width at half-maximum of rocking curves (ω-fwhm) is examined for the 0002 reflection and the 101̅1 reflection or the 101̅2 reflection. The film morphology of the samples is examined by top-view and cross-sectional scanning electron microscopy (SEM, Zeiss, Gemini 1, 3 kV). The film thickness of the samples is determined from cross-sectional SEM images and amounts to ∼40 nm for all AlN films and ∼400 nm for all GaN films. The polarity of the samples is determined by etching in aqueous KOH. Selected samples are etched in 1 wt % KOH solution at 70 °C. Quantitative analysis of oxygen incorporation is carried out by secondary ion mass spectrometry (SIMS, Cameca IMS 7f). Cross-sectional samples of the film stacks are extracted and thinned to electron transparency by the focused ion beam (FIB) technique using a FEI Helios Dual Beam system. Scanning transmission electron microscopy investigation using annular bright-field (ABF) and high-angle annular dark-field (HAADF) detectors is conducted on a probe C_s_-corrected JEOL NEOARM microscope operating at an accelerating voltage of 200 kV (cold FEG). Atomic imaging is improved by serial acquisition and nonrigid registration using the SmartAlign tool^33^ (HREM Research Inc.) and simple postfiltering by a radiance filter (lite version of DigitalMicrograph plug-in HREM-Filters Pro/Lite v.4.2.1, HREM Research Inc.). In addition to imaging of the layer and atomic structures, elemental analysis is conducted by energy-dispersive X-ray spectroscopy using a two wide-angle Si(Li)-drift detector system with an active area of 100 mm^2^ each. EELS spectra are recorded with a Gatan Enfinium ER spectrometer using spectrum imaging and in dual-mode enabling spectra deconvolution.

## Results and Discussion

### Film Morphology of AlN and GaN

**Figure 1:**
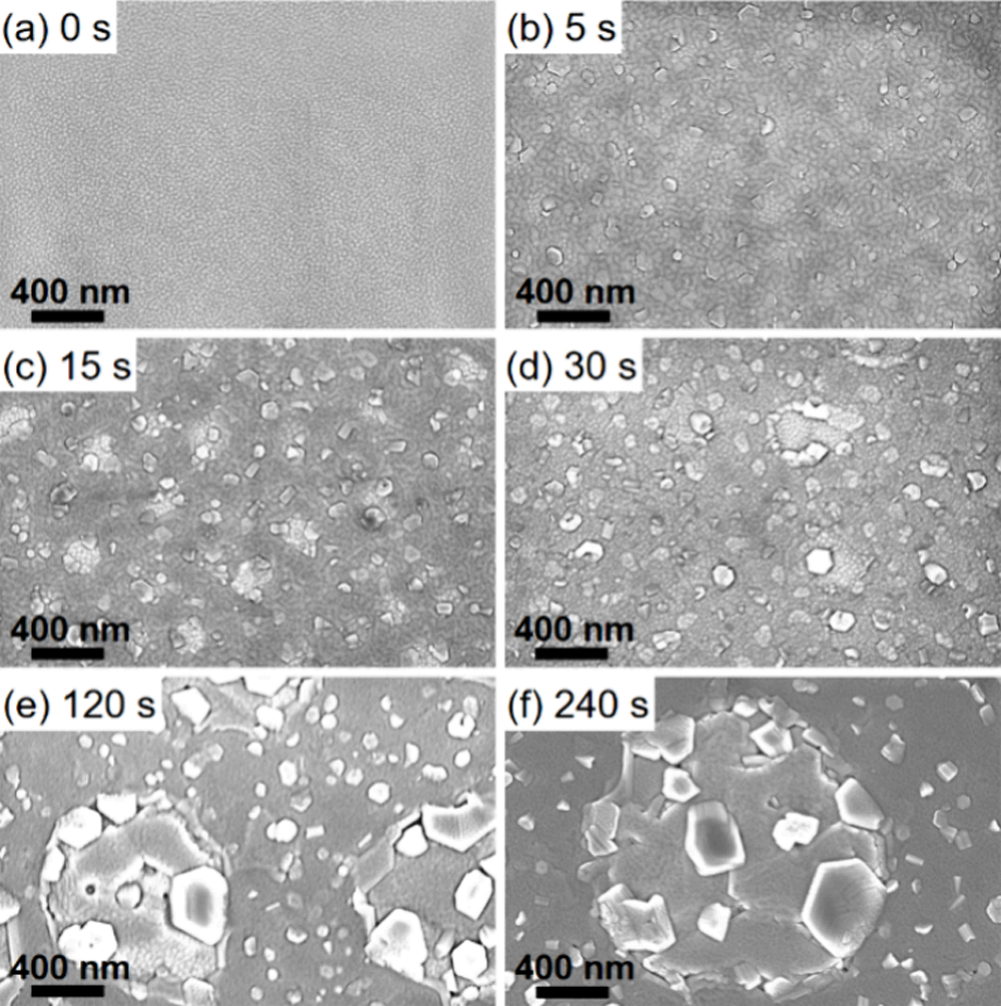
Top-view SEM images of AlN films grown on HF-etched Si(111) substrates with Al seed layer deposition times of (a) 0, (b) 5, (c) 15, (d) 30, (e) 120, and (f) 240 s.

The surface morphology of 40 nm thick AlN films grown on Si(111) substrates with varying Al seed layer growth times from 0 to 240 s is examined by top-view SEM images shown in Figure 1. The surface features give insight into the nucleation of the AlN films, as the seeding process at high temperatures is complex. Estimating the Al flux from the room temperature growth rate of 300 nm/h with an assumed Al density of 2.7 g/cm^3^ and an atomic mass of 27 g/mol, the Al flux amounts to ∼5 × 10^14^ atoms/cm^2^ s. However, the sticking coefficient and the diffusion rate of Al in Si drastically differ from the room temperature values.^34^ Additionally, a higher desorption rate at high temperatures needs to be considered.^35^ Thermal desorption increases exponentially with increasing temperature. At a certain temperature (∼200 °C)^36^ the increase in surface mobility is offset by the increase in thermal desorption. The AlN film deposited directly onto the Si(111) substrate without an Al seed layer (Figure 1a) exhibits a columnar but uniform morphology without distinct features. Starting at an Al seed layer growth time of 5 s (Figure 1b), lighter and darker areas appear in the SEM image showing dropletlike shapes emerging at the surface. Some of the features show the distinct hexagonal shape, which is associated with the hexagonal wurtzite crystal structure of AlN. Increasing the Al seed layer deposition time leads to the formation of flakelike surface structures that increase in number and size for longer Al seed layer growth times. The formation of droplet-shaped surface features becomes apparent for even longer Al seed layer growth times of 120 and 240 s (Figure 1e and f) and increase in size for longer Al seed layer growth time. These features may be due to Al droplets forming at the interface, as the growth temperature is higher than the melting point of pure Al and that of Al–Si alloys in a broad concentration range. Thus, we postulate that the sputtered Al melts on the heated Si surfaces and may form droplets to reduce surface tension, leading to the growth of randomly oriented hexagonal AlN columns on top of the droplets.

**Figure 2:**
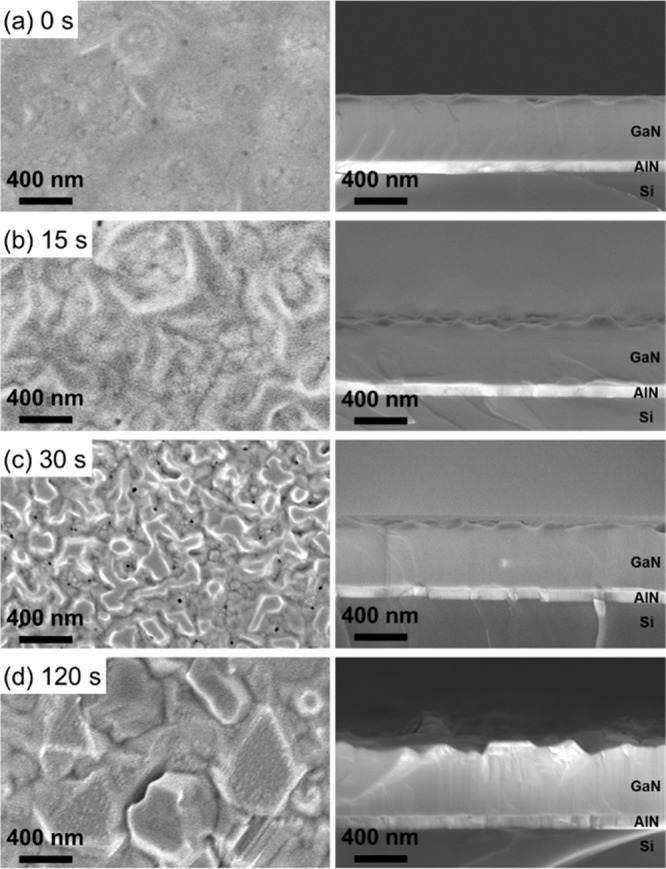
Top-view and cross-sectional SEM images of GaN/AlN films grown on HF-etched Si(111) substrates with Al seed layer deposition times of (a) 0, (b) 15, (c) 30, and (d) 120 s.

As-grown AlN films with Al seed layer growth times of 0–120 s are directly overgrown with 400 nm GaN in the same chamber. The morphology of the GaN films is examined by top-view and cross-sectional SEM images shown in Figure 2. The GaN/AlN/Si(111) film grown without an Al seed layer (Figure 2a) shows a relatively smooth surface but overall wavy film structure. For Al seed layer growth times of 15 and 30 s (Figure 2b and c), the GaN films exhibit an increasingly nonuniform and rough surface. Areas with different heights start to develop. For the GaN grown on AlN/Si with the longest Al seed layer growth time of 120 s (Figure 2 d), individual columns with different heights can be observed resulting in a rough, uneven surface. In the cross-sectional SEM images, no Al layer is observed at the AlN/Si interface. Although we assume local accumulation of Al at the AlN/Si interface, no continuous Al layer is formed. High diffusion rates of Al in Si at high substrate temperatures and high reactivity of Al with residual oxygen or nitrogen may prevent the formation of an Al layer. The flakelike surface features and footprints of the dropletlike features observed for AlN are overgrown by GaN. The increased roughness of AlN films grown with increasingly long Al seeding deposition time propagates through the GaN films, although distinct features of the AlN surface do not transfer to the GaN surface.

### Crystal Quality of AlN and GaN

**Figure 3:**
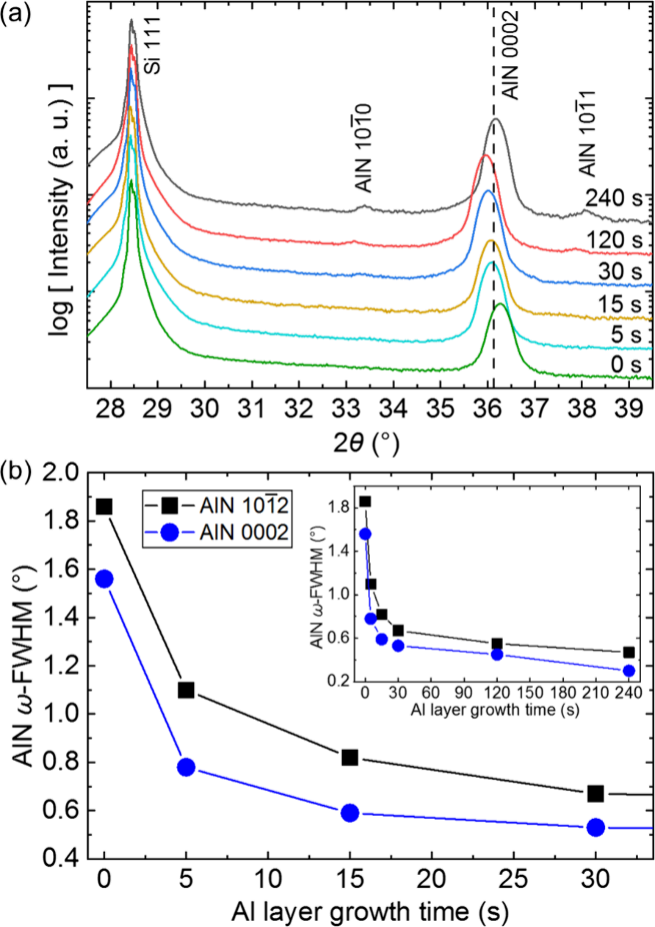
(a) XRD 2θ scans of AlN/Si(111) films with different Al seed layer growth times. The 2θ position of 0002 bulk AlN^37^ is marked with a dashed line. (b) ω-fwhm of the AlN 0002 and AlN 101̅2 reflection as a function of Al seed layer growth time. The inset shows Al growth times up until 240 s.

The crystal quality of AlN and GaN films grown with different Al seed layer deposition times is examined by XRD. For AlN, the 2θ scans (Figure 3 a) show a shift of the AlN 0002 reflection toward smaller 2θ values when the Al seed layer deposition time is increased from 0 to 120 s revealing increasingly enlarged *c*-axis lattice constants. Therefore, the AlN films exhibit increasing compressive strain with increasing Al seed layer deposition time. Strain in the films can originate from thermal and lattice mismatch to the substrate as well as the coalescence of AlN islands. The 2θ scan of the AlN film with an Al seed layer deposited for 240 s differs from that trend with the AlN 0002 reflection appearing at the 2θ position of 36.12° which corresponds to strain-free AlN.^37^ Relaxation of the lattice is the result of lattice defect formation, e.g., misfit dislocations. This results in a decoupling of film and substrate lattices above a critical thickness.^38,39^ If the Al seed layer is no longer coupled to the Si substrate, the strain is already relaxed at the Al seed layer resulting in strain-free growth conditions for the AlN nucleation layer. Additionally, the emergence of the AlN 101̅0 and AlN 101̅1 reflections is observed, indicating the formation of misoriented grains. Even for the longest Al seeding deposition time of 240 s, no Al reflections are observed in the 2θ scan. This is in agreement with the cross-sectional SEM images, indicating the absence of an Al layer at the interface. The crystal quality of the AlN films is evaluated by means of ω-fwhm of the AlN 0002 and AlN 101̅2 reflection which is depicted in Figure 3b as a function of the Al seed layer deposition time. Even a short deposition time (5 s) for the Al seeding improves the crystal quality of the AlN film significantly, reducing the ω-fwhm of the AlN 0002 and AlN 101̅2 reflections by half. Increasing the Al deposition time leads to further improvement of the crystal quality; however, the effect is limited. Depositing Al for more than 30 s leads to only a minor improvement in the crystal quality.

**Figure 4:**
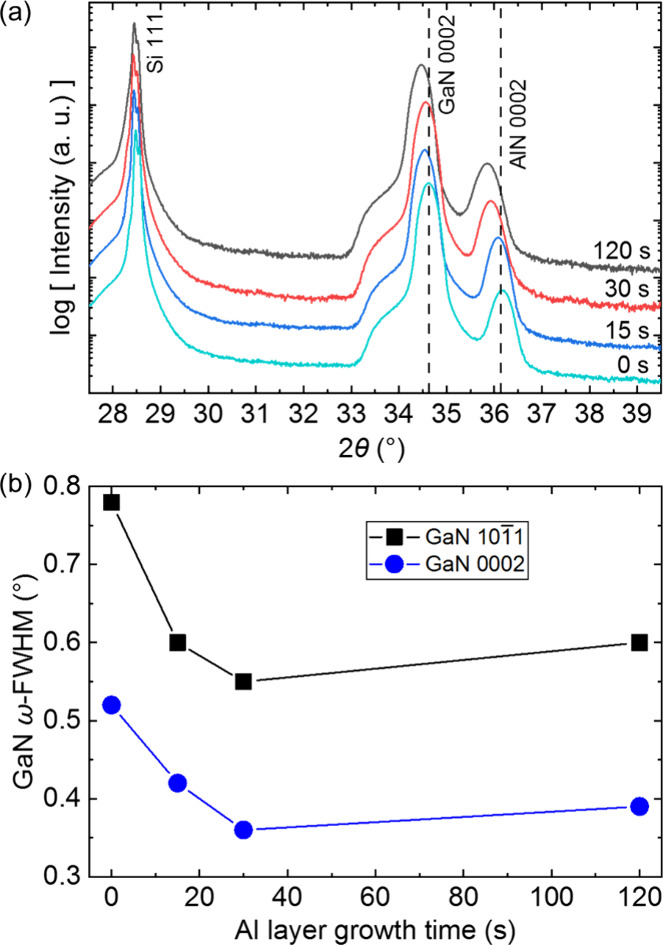
(a) XRD 2θ scans of GaN/AlN/Si(111) films with different Al seed layer growth times. The 2θ position of 0002 bulk GaN^46^ and AlN^37^ is marked as a reference. (b) ω-fwhm of the GaN 0002 and GaN 101̅1 reflection as a function of Al seed layer growth time.

The effect of different Al seed layer deposition times on the crystal structure of GaN grown on AlN/Si(111) templates is depicted in Figure 4. The GaN films follow the trend of the previously grown AlN films and show increasingly compressive strain with increasing Al seeding deposition time (Figure 4a). The improvement in crystal quality with increasing Al seed layer deposition time is likewise observed for GaN growth (Figure 4b). For 30 s Al layer growth time, the ω-fwhm of the GaN 0002 and GaN 101̅1 reflections decreases from 0.55° to 0.35° and 0.80° to 0.55°, respectively, indicating a significantly reduced threading dislocation density (TDD). To estimate the reduction of TDD, the formulas proposed by Dunn and Kogh utilizing the length of the Burgers vector *b* and the ω-fwhm β are used. The relationship is given by *D* = β^2^/4.35*b*^2^^40^ and used to calculate the values of screw-type TDD *D*_S_ and edge-type TDD *D*_E_ from XRD data. The tilt from mixed and screw dislocations can be directly derived from ω-scans of the GaN 0002 reflection since edge dislocations do not distort these planes as their Burgers vectors lie within.^41,42^ To calculate *D*_E_, β_twist_ can be extracted from the ω-fwhm of GaN 101̅1 reflections by assuming the nonlinear relationship β^2^ = (β_tilt_ cos *X*)^2^ + (β_twist_ sin *X*)^2^ with *X* being the angle between the pyramidal plane and basal plane.^43,44^ Twist is caused by edge and mixed dislocations; therefore, off-axis reflections occurring at high *X* angles are used to calculate the twist component as the ω-fwhm is dominated by in-plane twist.^45^ That gives a reduction in screw-type TDD from 7.9 × 10^9^ cm^–2^ to 3.2 × 10^9^ cm^–2^ and edge-type TDD from 5.1 × 10^10^ cm^–2^ to 2.4 × 10^10^ cm^–2^. However, the overall improvement in crystal quality is not as drastic compared to the improvement in the AlN nucleation layer. Since an AlN film with a good crystal quality improves the quality of the subsequently grown GaN film, a less drastic change can be expected. If dislocations intersect other dislocations, they can annihilate if the dislocations have Burgers vectors with opposite sign. If the film has a high number of dislocations per area, the room for improvement until it becomes statistically unlikely that dislocations intersect and annihilate is much greater. Although the trend of the increased compressive strain is continuous for Al seed layer growth times of up to 120 s, the ω-fwhm of the GaN 0002 and GaN 101̅1 reflection increases slightly going from 30 to 120 s Al seed layer deposition time, indicating a decrease in crystal quality. This might be due to the rough surface being a difficult nucleation site for GaN resulting in columnar growth with a broad range of tilt angles. Even nucleation on planes other than the *c*-plane could occur in the initial stages of GaN growth if the corresponding facets are exposed on the rough surface. As it appears that the benefits of an improved crystal quality of the AlN nucleation layer are outweighed by the detrimental effect of surface roughening on the GaN growth, an optimal Al seed layer deposition time can be determined. For lateral devices, however, smooth interfaces are crucial, while having outstanding crystalline quality in the nucleation and initial buffer layers can be compensated by growing thicker buffer layers. It can be expected that the threading dislocation density in the top layers will decrease once thick buffer layers are grown.

### Structural Analysis of AlN/Si(111) Interface and Film Polarity

**Figure 5:**
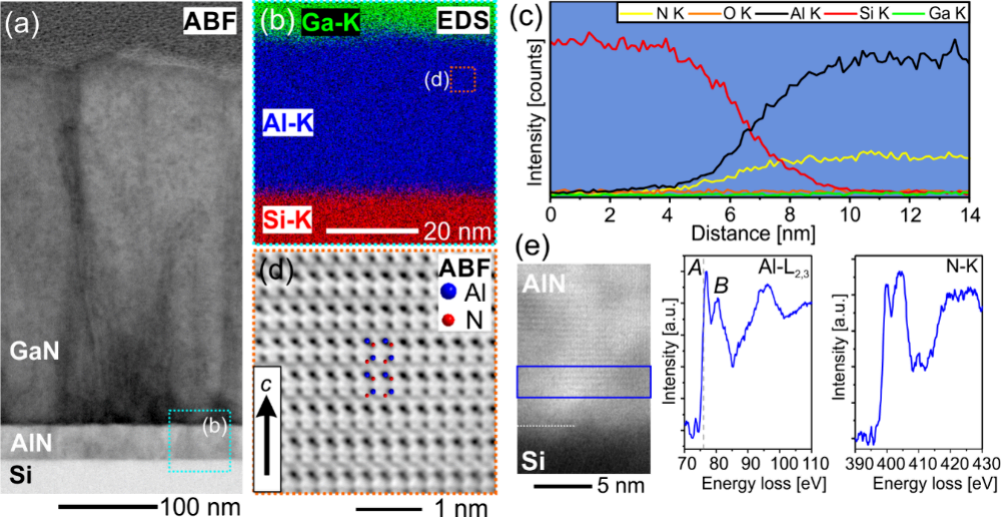
STEM examination of the AlN/Si nucleation layer grown without Al seeding. (a) ABF-STEM micrograph showing the complete layer stack. (b) EDS elemental map showing the signals of Si–K (red), Al–K (blue), and Ga–K (green). (c) EDS profile analysis across the AlN/Si interface. (d) Atomic resolution ABF-STEM image suggesting metal-polarity of the AlN layer. (e) EELS analysis of the AlN layer close to the interface showing the ELNES of Al–L_2,3_ and N–K core-loss transitions.

Nanoscale characterization of the atomic and chemical structures at the interfaces of AlN/Si stacks without Al seeding and 30 s of Al seeding is performed by STEM. EDS and EELS are used to study the AlN/Si interface in detail. The results for AlN growth without seeding are presented in Figure 5. The ABF-STEM micrograph in Figure 5a displays the film cross section featuring a 35 nm thin AlN layer with smooth interfaces to the Si substrate and the overgrown GaN film. Elemental mapping by EDS and the respective EDS profile across the AlN/Si interface shown in Figure 5b and c, respectively, further demonstrate chemically sharp interfaces without intermixing or amorphous interlayers as observed before.^12,47^ The atomic structure of the AlN nucleation layer is examined by ABF-STEM (Figure 5d) suggesting Al-polar growth along the wurtzite-type *c*-direction. However, despite the high in-plane ordering and the well-aligned *c*-axis texture of these AlN films, finding ideal imaging conditions poses a challenge due to local orientation inhomogeneities and superposition effects. Further, chemical analysis of the AlN film close to the Si interface is conducted by probing the energy-loss near edge structures (ELNES) of the Al–L_2,3_ and N–K core-loss transitions shown in Figure 5e. The analysis of the ELNES is in good agreement with literature data of AlN exemplifying the high-quality growth of the AlN nucleation layer.^12,48^

**Figure 6:**
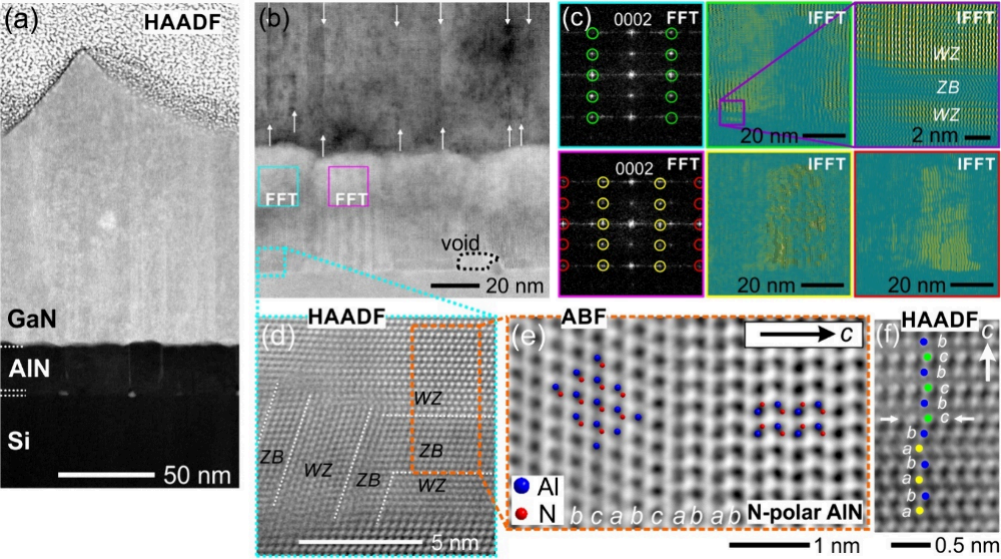
STEM examination of the AlN/seed/Si interface region grown with 30 s Al seed layer deposition time. (a) HAADF-STEM micrograph showing the complete layer stack. (b) Higher magnification ABF-STEM image of the rough GaN/AlN/seed layer system. Vertical two-dimensional defects originate at the GaN/AlN interface (white arrows). The seed layer shows voids, misoriented grains, and nonepitaxial domains identified by Fast Fourier Transfer (FFT) analysis (c) by retrieving the spatially distributed information on the highlighted reflections in the FFTs by calculation of the inverse FFT (IFFT). A loss of structural coherence along the film direction exhibiting ZB inclusions within the interlayer is observed (purple frame of c). (d) Atomic resolution HAADF-STEM image showing the crystalline structure of the interlayer containing regions of WZ and ZB stacking. (e) Atomic resolution ABF-STEM image showing the interface between the ZB and WZ-type domains in the seed layer suggesting N-polar nucleation of the AlN layer at this location. (f) HAADF-STEM image of a *c*-plane stacking fault in the overgrown AlN layer (periodicity: *ab***c***bc*).

**Figure 7:**
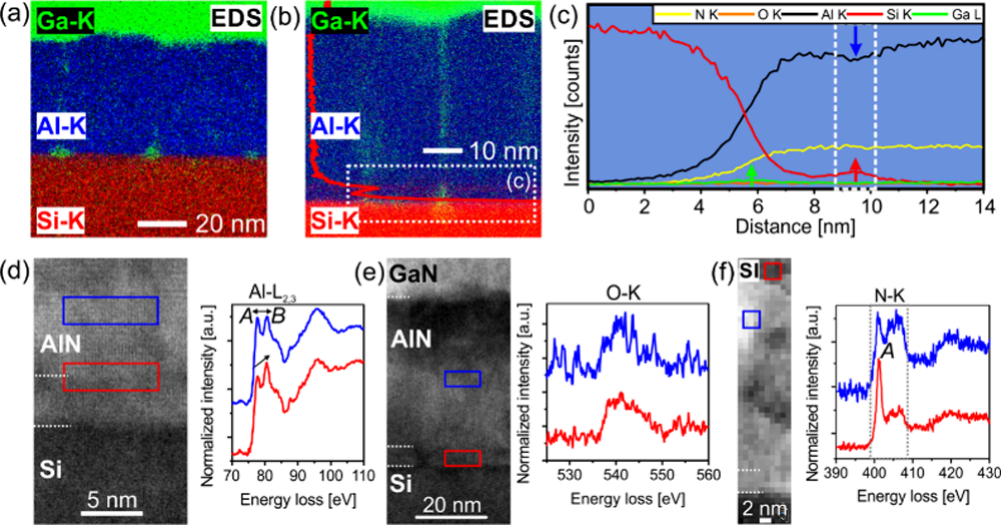
Spectroscopic studies of the AlN/seed/Si interface region grown with 30 s Al seed layer deposition time. (a), (b) EDS elemental maps (signals of Si–K: red, Al–K: blue, and Ga–K: green) showing grain boundary diffusion of Ga to the seed layer and diffusion of Si to the AlN/seed layer interface. (c) EDS profile analysis across the AlN/seed/Si interfaces showing the Si enrichment at the AlN/seed layer interface. (d) EELS analysis showing the change of Al-L_2,3_ ELNES at the AlN/interlayer interface (red frame) and ∼5 nm distant (blue frame). (e) EELS analysis of the oxygen content within the interlayer (red frame) and AlN film (blue frame) indicates a higher oxygen concentration at the interlayer. (f) EEL spectrum image mapping the total intensity of the N–K edge within the range of 399– 408 eV. Areas of low (red frame) and high intensity (blue frame) display large variation of the A peak intensity suggesting strong differences of the local chemical composition within the AlN film.

In contrast, growing AlN on Si with preceding Al seeding (30 s) leads to the formation of a structurally mixed interlayer. The HAADF-STEM image presented in Figure 6a shows the growth of a ∼4 nm thin interlayer and a ∼45 nm AlN film with higher surface roughness. In comparison to the film stack without Al seeding, the GaN film grown using Al seeding in the process flow shows a large number of vertical defects which may be low-angle grain boundaries originating at the rough AlN nucleation layer (Figure 6b). The change in the defect structure observed in the GaN layer seems directly related to the mixed seed layer featuring misoriented grains, larger voids, and the loss of structural coherence featuring inclusions with cubic stacking (Figure 6b and d). The ill-defined structure of the seed layer impacts the crystal quality of the overgrown AlN layer resulting in the identification of structural defects, e.g., stacking faults and the partial violation of the epitaxial nature observed for individual columnar grains of AlN (Figure 6c). These structural features of the AlN and seed layer domains result in a locally highly defective mosaic crystal acting as pathways for diffusion. This contrasts the more homogeneous mosaic crystal observed without Al seeding and interlayer formation. In the HAADF-STEM image shown in Figure 7a, droplets with brighter Z-contrast are observed at the seed layer/Si interface and grain boundaries within the AlN nucleation layer. EDS analysis reveals the presence of Ga and Si in these regions which concludes on diffusion along these defective regions. The elemental maps and the EDS profile are provided in Figure 7a–c. The overall oxygen content in the AlN nucleation layer is examined to be slightly higher at the seed layer (O K map Figure 7e) and the AlN layer (oxygen ∼14 at. %) when compared to the quantified signals of O–K to Al–K and N–K to the AlN nucleation layer without the interlayer (oxygen ∼3 at. %). In conclusion, the chemical composition of the defect-rich seed layer is identified as oxygen-rich AlN which is partially capped with a Si-rich monolayer.

Further, EELS studies of the chemical structure of the seed layer and the AlN thin film are provided in Figure 7d–f. The comparison of the ELNES of the Al-L_2,3_ edge at the AlN/interlayer interface shows a similar intensity distribution for peaks labeled “A” and “B” in the AlN film. In contrast, the Al-L_2,3_ ELNES recorded for the AlN film grown without the interlayer shows a stronger intensity of the A peak. In addition, a reduction of the B peak intensity is observed in the AlN layer compared to the interlayer. The higher intensity of the B peak at the interlayer interface may be explained by enhanced octahedral coordination due to oxidation, showing similarity to reported data for alumina.^49−51^ Indeed, the acquired O–K signals (compare the signal/noise ratios of the O–K signal in the interlayer and AlN film, Figure 7e) match with the increased contribution of oxygen as demonstrated by the EDS signals recorded from the interlayer. Moreover, further discontinuities in the chemical structure of the AlN film are observed from mapping the intensity of the recorded N–K signal as demonstrated in Figure 7f. Here, brighter and darker regions are identified and related to strong differences in the A peak (∼401 eV) intensity pointing toward growth discontinuities related to nitrogen defects.^52,53^

The revealed complex chemical structure of the seed layer interfaces enriched with Si and O provides a very different nucleation environment for the AlN film than a pure homogeneous Si(111) surface. The introduction of oxygen and silicon-rich layers into AlN films is often investigated as a strategy to invert the polarization of wurtzite-type materials from N- to metal-polarity^54^ and vice versa.^55^ Hence, the local atomic structure of the interlayer and the polarity of the AlN film nucleating at the interface are examined with atomic resolution STEM. A clear structural inhomogeneity is identified showing domains with zinc blende (ZB) and wurtzite (WZ) stacking along both horizontal and vertical directions (Figure 6d). The unit cell termination of the AlN layer nucleating on the horizontal ZB domain shows N-polarity along the *c*-direction indicated in the ABF-STEM image showing the interface of the ZB domain (cubic stacking *abc*) and the WZ-AlN film (hexagonal stacking *ab*) in Figure 6e. The analysis of an adjacent grain nucleating on the vertically staggered ZB/WZ interlayer domain, however, suggests the nucleation of an Al-polar domain featuring a *c*-plane stacking fault (*ab***c***b***c**) (Figure 6f), demonstrating the presence of an AlN layer with mixed polarity. However, STEM investigation of the overgrown GaN layer could not unambiguously disclose the unit cell polarity due to the high number of vertical defects, locally distorting the lattice. Hence, a global change in the GaN film polarity cannot be derived based on STEM images.

**Figure 8:**
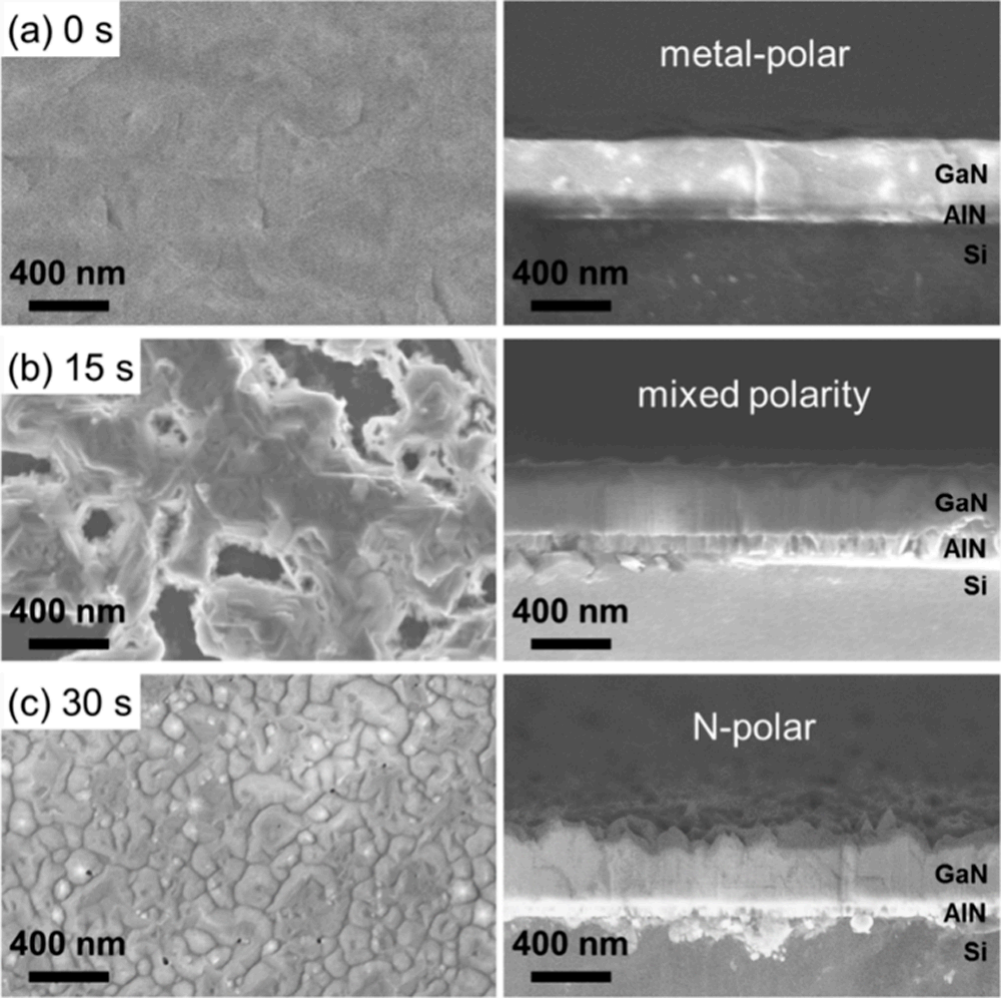
Top-view and cross-sectional SEM images of KOH-etched GaN/AlN/Si(111) stacks with different Al seed layer deposition times of (a) 0, (b) 15, and (c) 30 s. The films depicted in (a) and (b) have been etched for 30 min and the film depicted in (c) for 5 min due to the difference in etch rate for the different polarities. The polarity switches from metal-polar to N-polar if Al is sputtered for sufficient time.

To determine the polarity of the GaN layers in dependence on Al seeding, etching experiments are performed. The polarity of the III-nitrides can be revealed on a macroscopic scale by selective and anisotropic wet etching using an aqueous KOH solution. This etch is known to preferentially target N-polar surfaces of GaN, while only defect sites of Ga-polar surfaces are attacked.^56,57^Figure 8 shows top-view and cross-sectional SEM images of KOH-etched GaN/AlN/Si(111) stacks with different Al seed layer deposition and KOH etch times. Without Al seeding, the surface remains smooth after etching, showing that the GaN is Ga-polar (Figure 8a). Intermediate Al seed layer deposition times (15 s) resulted in mixed polarity films as shown in the SEM images of Figure 8b. Parts of the surface area are smooth, while other areas are predominantly etched. This is a result of the large difference in etch rates of Ga-polar films compared to N-polar films. While the Ga-polar part of the film is mostly unaffected by the KOH etch, the N-polar part of the film is rapidly etched away. GaN grown on AlN templates with longer Al seed layer deposition times (30 s) exhibit a rough pyramidal surface structure after KOH etching indicative of N-polar GaN (Figure 8c).^58,59^ Additionally, the expected etch rates are consistent with the surface structure observations. While the GaN/AlN film without an Al seed layer exhibits KOH etch rates of less than 1 nm/min, the sample with a 30 s sputtered Al seed layer exhibits KOH etch rates exceeding 40 nm/min. In conclusion, the combination of STEM and etching experiments demonstrates the inversion of polarity by the integration of an Al seed layer for 30 s from metal-polarity to N-polarity in AlN and GaN films. These results provide evidence of the possibility to control the polarity of GaN films by MSE via the integration of Al at the Si interface.

The change in polarity is also accompanied by a change in oxygen incorporation. SIMS measurements reveal oxygen levels of ∼10^17^ cm^–3^ for Ga-polar GaN and oxygen levels of ∼10^18^ cm^–3^ for N-polar GaN. The increased incorporation of oxygen in the N-polar film matches the EELS and EDS analysis of the oxygen content (Figure 7). The SIMS result is consistent with previous observations in GaN epitaxy and can be explained by the growth kinetics in terms of the specific structural configurations.^30,31^ In the case of Ga-face GaN, the establishment of the Ga bilayer hinders oxygen incorporation. The formation of such a barrier is not possible on N-face GaN.

## Conclusions

The film morphology, crystal quality, and polarity of GaN/AlN film stacks grown on Si(111) substrates are examined as a function of Al seed layer deposition time. Depositing Al at the interface leads to an initially drastic improvement of crystal quality for both AlN and GaN overgrowth, but the effect saturates for Al deposition times longer than 30 s. The surface morphology of AlN films becomes increasingly rough with increasing Al seed layer deposition time and the effect transfers to subsequently grown GaN films. The benefits of an improved crystal quality of the AlN nucleation layer are outweighed by the detrimental effect of surface roughening on the GaN growth. Moreover, Al integration at the Si interface can be used to switch the polarity of the GaN/AlN stacks. Without the deposition of Al at the interface, the films are metal-polar. Aiming for lateral HEMT devices as an application, metal-polar GaN/AlN film stacks with a smooth surface grown without an Al seed layer may be the preferred choice. Depositing the Al seed layer for a sufficiently long time leads to an inversion in polarity. A polycrystalline oxygen-rich AlN interlayer capped by an atomically thin layer rich in Si forms at the interface and is proposed to induce N-polar growth.
